# Sleep quality and falls in middle-aged and older adults: ELSI-Brazil study

**DOI:** 10.1590/1980-220X-REEUSP-2024-0027en

**Published:** 2024-07-05

**Authors:** André Pereira dos Santos, Jéssica Fernanda Correa Cordeiro, Pedro Pugliesi Abdalla, Lucimere Bohn, Emerson Sebastião, Leonardo Santos Lopes da Silva, Márcio Fernando Tasinafo, Ana Cláudia Rossini Venturini, Alynne Christian Ribeiro Andaki, Edmar Lacerda Mendes, Pablo Jorge Marcos-Pardo, Jorge Mota, Dalmo Roberto Lopes Machado

**Affiliations:** 1 Universidade do Porto, Faculdade de Desporto, Centro de Investigação em Atividade Física, Saúde e Lazer, Porto, Portugal.; 2 Universidade de São Paulo, Escola de Educação Física e Esporte de Ribeirão Preto, Ribeirão Preto, Brasil.; 3 Universidade de São Paulo, Escola de Enfermagem de Ribeirão Preto, Ribeirão Preto, Brasil.; 4 Universidade de São Paulo, Escola de Educação Física e Esporte de Ribeirão Preto, Grupo de Estudo e Pesquisa em Antropometria, Treinamento e Esporte, Ribeirão Preto, Brasil.; 5 Universidade do Porto, Faculdade de Desporto e Laboratório de Investigação Integrativa e Translacional em Saúde da População, Centro de Investigação em Atividade Física, Saúde e Lazer, Porto, Portugal.; 6 Universidade Lusófona, Centro de Investigação em Desporto, Educação Física, Exercício e Saúde, Porto, Portugal.; 7 University of Illinois Urbana-Champaign, Department of Health and Kinesiology, United States.; 8 Universidade Federal do Triângulo Mineiro, Departamento de Ciências do Esporte, Uberaba, Brasil.; 9 Universidad de Almería, Departamento de Educación, SPORT Research Group (CTS-1024), Centro de Investigación Para el Bienestar y la Inclusión Social, Almería, Spain.; 10 Universidade do Algarve, Escola Superior de Educação e Comunicação, Penha Campus, Faro, Portugal.

**Keywords:** Adult, Aged, Accidental Falls, Sleep Hygiene, Sleep Quality, Adulto, Idoso, Acidentes por Quedas, Higiene do Sono, Qualidade do Sono, Adulto, Anciano, Accidentes por Caídas, Higiene del Sueño, Calidad del Sueño

## Abstract

**Objective:**

To verify the association between low self-reported sleep quality (LSQ) and fall in middle-aged and older adults every half-decade of life.

**Method:**

A cross-sectional study was conducted using data from the first wave (2015–2016) of the Brazilian Longitudinal Study of Aging (ELSI-Brazil), which is nationally representative. The sample consisted of 8,950 participants who were allocated into eight age groups: 50–54, 55–59, 60–64, 65–69, 70–74, 75–79, 80–84, and ≥ 85 years. The questionnaires used included self-reported sleep quality and the International Physical Activity Questionnaire short version. Fisher’s exact test followed by binary logistic regression analysis was performed to identify the odds ratio of sleep quality for fall occurrence, controlled for confounding variables.

**Results:**

Individuals aged 50–105 years (63.6 ± 10.2 years), 57.0% females and 43.0% males, participated in this study. Overall, 21.5% of participants experienced at least one fall. The relative frequency of participants classified as having high or LSQ remained constant across each half-decade of life. The LSQ exhibited a statistically significant OR (p < 0.05) for falls across age groups up to 84, even after accounting for confounding variables.

**Conclusion:**

LSQ is significantly associated with an increased occurrence of fall in adults aged >50 years, but not for ≥ 85 years regardless of sex and physical activity level.

## RESUMO

**Objetivo:**

Verificar a associação entre baixa qualidade do sono autorrelatada (BQS) e quedas em adultos de meia-idade e idosos a cada meia década de vida.

**Método:**

Um estudo transversal foi conduzido utilizando dados da primeira onda (2015–2016) do Estudo Longitudinal Brasileiro do Envelhecimento (ELSI-Brasil), que é nacionalmente representativo. A população consistiu em 8.950 participantes que foram alocados em oito grupos etários: 50–54, 55–59, 60–64, 65–69, 70–74, 75–79, 80–84 e ≥ 85 anos. Os questionários utilizados incluíram qualidade do sono autorrelatada e o Questionário Internacional de Atividade Física versão curta. O teste exato de Fisher seguido pela análise de regressão logística binária foi conduzida para identificar a razão de chances da BQS para ocorrência de queda, controlando por variáveis de confusão.

**Resultados:**

Pessoas com idades entre 50 e 105 anos (63,6 ± 10,2 anos), sendo 57,0% do sexo feminino e 43,0% do sexo masculino, participaram deste estudo. No geral, 21,5% dos participantes experimentaram pelo menos uma queda. A frequência relativa de participantes classificados como tendo BQS ou alta permaneceu constante em cada meia década de vida. A BQS exibiu uma OR (p < 0,05) notável para quedas em grupos etários até 84 anos, mesmo após o ajuste para variáveis de confusão.

**Conclusão:**

A BQS está significativamente associada a uma maior ocorrência de queda em adultos com mais de 50 anos, mas não para ≥ 85 anos, independentemente do sexo e do nível de atividade física.

## RESUMEN

**Objetivo:**

Verificar la asociación entre la baja calidad del sueño autorreportada (BCS) y las caídas en adultos de mediana edad y mayores cada media década de vida.

**Método:**

Se realizó un estudio transversal utilizando datos de la primera oleada (2015–2016) del Estudio Longitudinal Brasileño sobre el Envejecimiento (ELSI-Brasil), que es representativo a nivel nacional. La muestra consistió en 8,950 participantes que fueron asignados a ocho grupos de edad: 50–54, 55–59, 60–64, 65–69, 70–74, 75–79, 80–84 y ≥ 85 años. Los cuestionarios utilizados incluyeron calidad del sueño autorreportada y el Cuestionario Internacional de Actividad Física versión corta. Se realizó una prueba exacta de Fisher seguida por un análisis de regresión logística binaria para identificar la razón de probabilidades de la calidad del sueño para la ocurrencia de caídas, controlando las variables de confusión.

**Resultados:**

Participaron en este estudio individuos con edades entre 50 y 105 años (63,6 ± 10,2 años), siendo el 57,0% mujeres y el 43,0% hombres. En general, el 21,5% de los participantes experimentaron al menos una caída. La frecuencia relativa de participantes clasificados como con alta calidad de sueño o baja calidad de sueño (BCS) se mantuvo constante a lo largo de cada media década de vida. La BCS mostró un OR (p < 0,05) notable para las caídas en grupos de edad hasta los 84 años, incluso después de ajustar por variables de confusión.

**Conclusión:**

La BCS está significativamente asociada con una mayor ocurrencia de caídas en adultos mayores de 50 años, pero no para ≥ 85 años, independientemente del sexo y del nivel de actividad física.

## INTRODUCTION

The aging process is distinguished by an intricate interplay of numerous physiological changes and the accrual of chronic conditions over time, affecting the overall well-being and functional capacities of older adults^(1)^. Worldwide, demographics are undergoing a significant transformation marked by a rising aging population. This trend is pervasive, as every nation witnesses an escalation in both the absolute number and relative proportion of older adults in their demographic structure^(2)^. Projections from the World Health Organization suggest that, by the year 2050, approximately 80% of the older population will be concentrated in low- and middle-income countries^(2)^. As per the World Health Organization’s forecasts, the global population aged 60 and above is expected to reach 2 billion by 2050, comprising a substantial one-fifth of the global population^(2)^. Additionally, statistics from the Ministry of Health indicate that, in the year 2016, Brazil held the fifth position globally in terms of its older population^(3)^. Forecasts indicate that by 2030, the number of older adults in Brazil will exceed the total count of children aged zero to 14^(3)^.

Such growth comes with significant individual, societal, economic, and medical challenges. For instance, there is evidence that about 80% of older adults present with at least one chronic condition, and 50% have at least two^(4)^. Further, a recent report revealed that nearly 20% of older adults reported some level of physical function problems^(5)^. Falls are another concern among older adults and represent a global public health problem. This is based on its global prevalence and consequences (e.g., severe health loss, including death)^(6)^. For instance, a recent review compiling data from 104 studies with a total sample of 36,740,590 revealed the fall prevalence in older adults of the world was 26.5%. In addition, fall-related injuries can be fatal or non-fatal and physical or psychological, which leads to a reduction in the ability to perform basic and instrumental activities of daily living - negatively impacting the quality of life^(7)^.

Accidental falls cause substantial morbidity, disability, and mortality across all age groups^(8)^, with older adults being particularly vulnerable, as falls are a leading cause of death in this population^(9)^. Research indicates that approximately one-third of adults aged 65 and above experience a fall at least once per year, with half of these cases being recurrent^(10)^. This can lead to a fear of falling, causing individuals to limit physical activities to prevent future fall^(11)^. The resulting morbidity from falls can also lead to comorbid conditions, placing significant burdens on healthcare systems^(12)^. Understanding fall factors is vital for developing effective strategies aimed at reducing the occurrence of falls. By identifying and addressing these factors, targeted interventions can be implemented to enhance fall prevention efforts and promote the safety and well-being of the older adult population.

Recently, the association between sleep quality and falls has garnered increasing interest in scientific research. Study have demonstrated that low self-reported sleep quality (LSQ) can adversely affect various physiological aspects that contribute to postural stability and neuromuscular function, particularly in middle-aged individuals and those above 60 years of age, increasing falls^(13)^. Sleep quality plays a pivotal role in regulating vital functions of the organism, including cellular recovery and repair, memory consolidation, and hormonal regulation^(14,15)^. Nevertheless, as individuals age, many experience alterations in sleep patterns, such as reduced sleep duration, sleep fragmentation, and decreased sleep efficiency. These changes may lead to decreased muscle strength, impaired motor coordination, and compromised balance, rendering individuals more susceptible to falls and subsequent injuries. Importantly, fall prevention becomes a crucial aspect of promoting healthy aging, especially from middle age onwards and particularly during old age. Investigating the relationship between sleep quality and falls holds paramount importance in the fields of public health and preventive medicine. Studies that explore factors associated with fall occurrence (e.g., sleep quality) form the basis for intervention proposals focused on promoting healthy sleep habits and improving sleep quality in middle-aged and older adults. In this context, understanding the influence of sleep quality on the occurrence of falls, along with intervention approaches, can significantly reduce falls and mitigate severe consequences, such as fractures and hospitalizations. In this context, we hypothesize that LSQ is correlated with a heightened occurrence of falls in individuals in their 50s and subsequent years of life, even when accounting for confounding variables such as sex and levels of physical activity. Therefore, this study aimed to verify the association between LSQ and fall in middle-aged and older adults every half-decade of life.

## METHOD

### ELSI-Brazil

The ELSI (Estudo Longitudinal da Saúde do Idoso) survey was initiated in 2015 in Brazil. ELSI is a longitudinal study strategically crafted to evaluate the health and aging aspects of the older Brazilian population. The survey has been structured for triennial implementation, intending to offer comprehensive insights into health conditions, lifestyles, and diverse factors influencing the aging process over an extended timeframe. Further information about ELSI-Brazil can be accessed on the research homepage (https://elsi.cpqrr.fiocruz.br/en/home-english/).

### Study Design

In this cross-sectional study, the data from the first wave of the ELSI-Brazil in 2015-2016 was considered. To ensure a representative sample encompassing urban and rural areas of small, medium, and large municipalities, ELSI-Brazil adopted a multistage stratified cluster sampling design. Municipalities were divided into four strata based on population size. For the first three strata (municipalities with up to 750,000 inhabitants), the sample selection involved three stages: municipality, census tract, and household. In the fourth stratum, comprising the largest municipalities, the sample was chosen in two stages: census tract and household. Households were systematically chosen with a jump of four houses after an interview or three unsuccessful contact attempts. However, this systematic jump was not applied in cases of refusal or ineligibility, such as when no resident aged 50 years or over was present, the household was vacant, or it was a collective residence (e.g., pension, asylum, republic, shelter, or hostel). Additionally, if the interviewee had a disability preventing them from completing the questionnaire without a substitute informant (proxy), the systematic jump was also skipped, and the interviewer proceeded to the next household using the right-hand rule. All residents aged 50 years and over in the selected households, including those with disabilities, bedridden participants, and wheelchair users, were eligible for participation in the research. The ELSI-Brazil study is nationally representative, encompassing people aged 50 years or older, residing in 70 municipalities across the 5 Brazilian regions. Further information on the ELSI Brazil’s sample and its national representativeness has been previously published^(16)^. Other details can also be seen on the research homepage (http://elsi.cpqrr.fiocruz.br/en/home-english/). ELSI-Brazil was approved by the ethics board of FIOCRUZ, Minas Gerais. Participants signed separate informed consent forms for the interviews and physical measurements, and access to administrative records.

### Data Collection

The questionnaires used in ELSI-Brazil were deemed correct and validated for the Brazilian population aged over 50 years, namely:

### Personal Characteristics

In face-to-face interviews, personal characteristics such as age (in years) and sex (male; female) were assessed. For the purpose of this study, sex was considered a confounding variable.

### Falls

The primary objective of this investigation revolved around the dependent variable, which entailed identifying the occurrence of a minimum of one fall. This data was collected through the following inquiry: “Have you experienced any falls within the past 12 months?” Thus, the response option is dichotomous, “yes” or “no”. For this study, falls were precisely defined as “unintentional displacements of the body to a lower level than the initial position, characterized by an inability to promptly regain stability, attributed to multifactorial circumstances compromising balance^”(17)^.

### Self-Reported Sleep Quality

Self-reported sleep quality was evaluated by interviewers during the home visit, utilizing a single question: “How do you assess your sleep quality?” Responses were recorded using a Likert scale, offering options such as “very good,” “good,” “regular,” “bad,” and “very bad.” For the specific aims of this investigation, self-reported sleep quality was dichotomously categorized. Participants who responded with “very good” and “good” were classified as having high self-reported sleep quality, while those who selected “regular,” “bad,” and “very bad” were categorized as experiencing low self-reported sleep quality (LSQ).

### Physical Activity Level

Physical activity was measured using the Brazilian-validated short version of the International Physical Activity Questionnaire (IPAQ-SV)^(18)^. The IPAQ-SV was administered to participants to assess their physical activity level in the week leading up to the interview. This instrument comprehensively evaluates various domains and intensities of physical activity, including walking and sitting time, which participants engage in as part of their daily routines. The IPAQ categorizes and conceptualizes these activities as follows: (a) Sedentary: Participants who do not engage in any physical activity for a minimum of 10 continuous minutes during the week. (b) Insufficiently active: Participants who perform physical activities for a minimum of 10 continuous minutes per week, but not enough to be classified as active. (c) Active: This category includes participants who meet the following recommendations: (a) vigorous physical activity for at least 3 days per week, with each session lasting at least 20 minutes; (b) moderate activity or walking for at least 5 days per week, with each session lasting at least 30 minutes; (c) any additional activity for at least 5 days per week, with a cumulative duration of at least 150 minutes per week. (d) Very active: This group comprises participants who meet the following recommendations: (a) engaging in vigorous activity for at least 5 days per week, with each session lasting at least 30 minutes; (b) participating in vigorous activity for at least 3 days per week, with each session lasting at least 20 minutes, and engaging in moderate activity and/or walking for at least 5 days per week, with each session lasting at least 30 minutes. Participants were divided into two groups: sedentary (including both the sedentary and insufficiently active categories) and active (encompassing the active and very active categories)^(19)^. For the purpose of this study, physical activity level was considered a confounding variable.

### Statistical Analysis

Following the acquisition of ELSI-Brazil data in CSV format, the dataset was imported into STATA software version 16.0 (Stata Corporation, College Station, Texas, USA). Subsequently, the dataset was converted to Microsoft Excel® spreadsheet format. Data coding was executed independently by two researchers, and validation was conducted through double-checking in Microsoft Excel® to minimize the potential for bias during data tabulation. The variables, encompassing sex (male [code = 0]; female [code = 1]), age group (50 to 54; 55 to 59; 60 to 64; 65 to 69; 70 to 74; 75 to 79; 80 to 84; and ≥ 85 years), falls (no [code = 0]; yes [code=1]), self-reported sleep quality (high [code=0]; low [code = 1]), and physical activity level (active [code = 0]; sedentary [code = 1]), were presented in terms of absolute (n) and relative (%) frequencies. To examine the association between falls (dependent variable) and self-reported sleep quality (predictor variable) within each group, Fisher’s exact test was employed. Statistical analyses were performed using the SPSS® version 20.0 program. For evaluating the OR of participants with LSQ concerning falls, binary logistic regression was conducted. Additionally, the binary logistic regression model was adjusted to include sex and physical activity level as confounding variables to assess their potential influence on the relationship between LSQ and falls. Significance level of α = 5% was applied to assess the statistical significance of the findings.

## RESULTS

**Figure 1:**
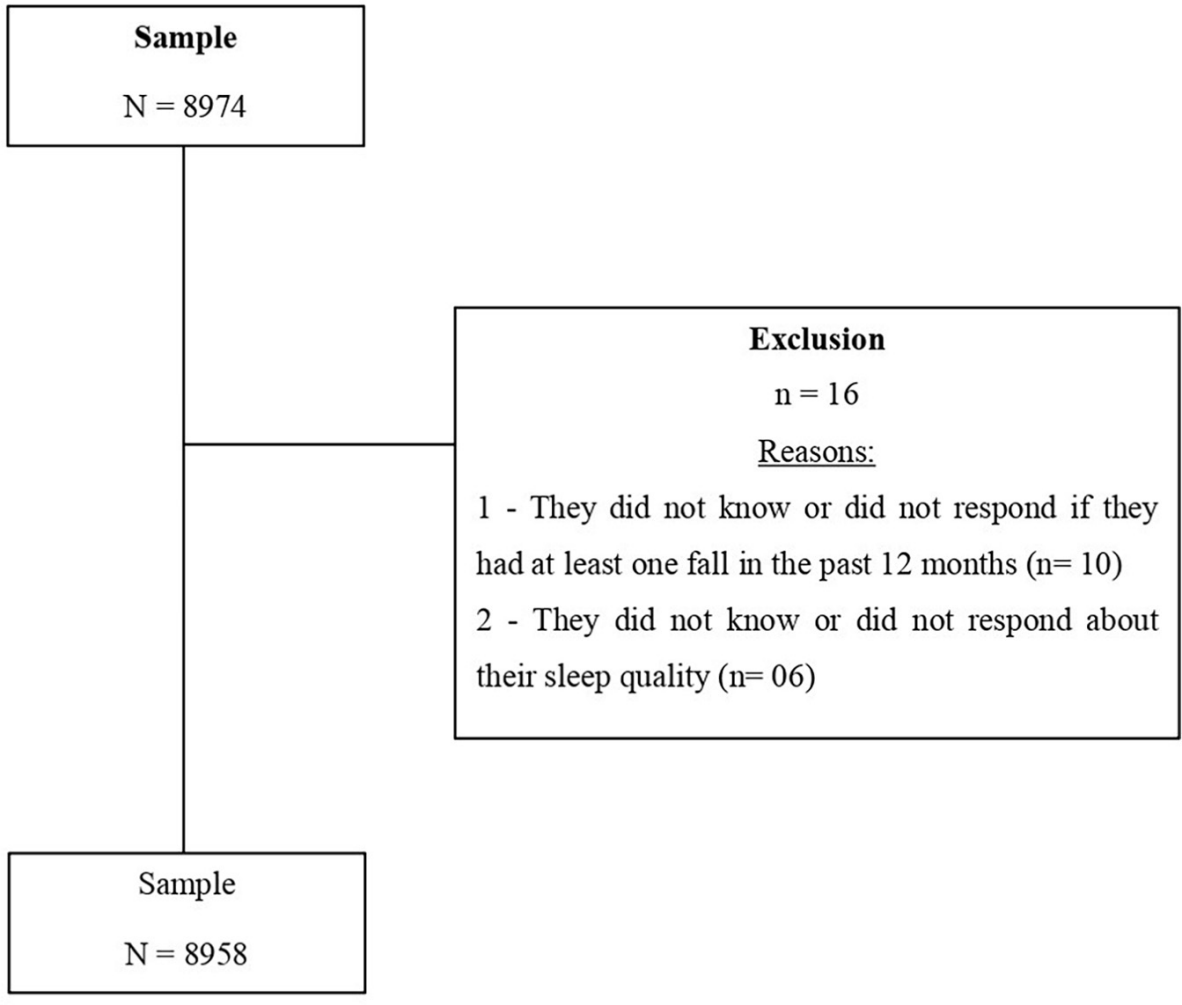
Study flowchart.

Figure 1 describes the flowchart of the participants throughout the study. A total of 16 were excluded according to the reasons below.

Our analytical sample consisted of 8,950 participants aged 50 to 105 years, with a mean age of 63.6 ± 10.2 years. Among them, 3,884 were males with a mean age of 62.7 ± 10.0 years, and 5,066 were females with a mean age of 64.3 ± 10.3 years. In Table 1, a consistent homogeneity of falls in the last year was observed across all age groups, ranging from 21.5% to 23.6%. Regarding self-reported sleep quality, the relative frequency of participants classified as having high or LSQ remained constant across each half-decade of life. In terms of physical activity level, an exponential decrease in the number of participants classified as active was observed over each half-decade of life.

**Table 1:** Falls, sex, self-reported sleep quality, and physical activity level classification by age grouping – Brazil 2015–2016.

Age grouping (years)									
Variables (n; %)	Clas	50–54 (n = 2097)	55–59 (n = 1679)	60–64 (n = 1476)	65–69(n = 1233)	70–74 (n = 957)	75–79 (n = 756)	80–84 (n = 422)	≥ 85 (n = 330)
**Falls**	**No**	1616 (77.1)	1282 (76.4)	1135 (76.9)	968 (78.5)	738 (77.1)	572 (75.7)	325 (77.0)	252 (76.4)
	**Yes**	481 (22.9)	397 (23.6)	341 (23.1)	265 (21.5)	219 (22.9)	184 (24.3)	97 (23.0)	78 (23.6)
**Sex**	**Male**	1007 (48.0)	811 (48.3)	618 (41.9)	490 (39.7)	379 (39.6)	294 (38.9)	150 (35.5)	135 (40.9)
	**Female**	1090 (52.0)	868 (51.7)	858 (58.1)	743 (60.3)	578 (60.4)	462 (61.1)	272 (64.5)	195 (59.1)
**Self-reported sleep quality**	**High**	1133 (54.0)	933 (55.6)	785 (53.2)	692 (56.1)	515 (53.8)	412 (54.5)	229 (54.3)	169 (51.2)
	**Low**	964 (46.0)	746 (44.4)	691 (46.8)	541 (43.9)	442 (46.2)	344 (45.5)	193 (45.7)	161 (48.8)
**Physical activity level**	**Active**	1489 (71.0)	1166 (69.4)	1000 (67.8)	793 (64.3)	575 (60.1)	402 (53.2)	197 (46.7)	104 (31.5)
	**Sedentary**	608 (29.0)	513 (30.6)	476 (32.2)	440 (35.7)	382 (39.9)	354 (46.8)	225 (53.3)	226 (68.5)

Legend: n = number; % = percentage; Clas= Classification.

Font: ELSI-Brazil.

**Figure 2:**
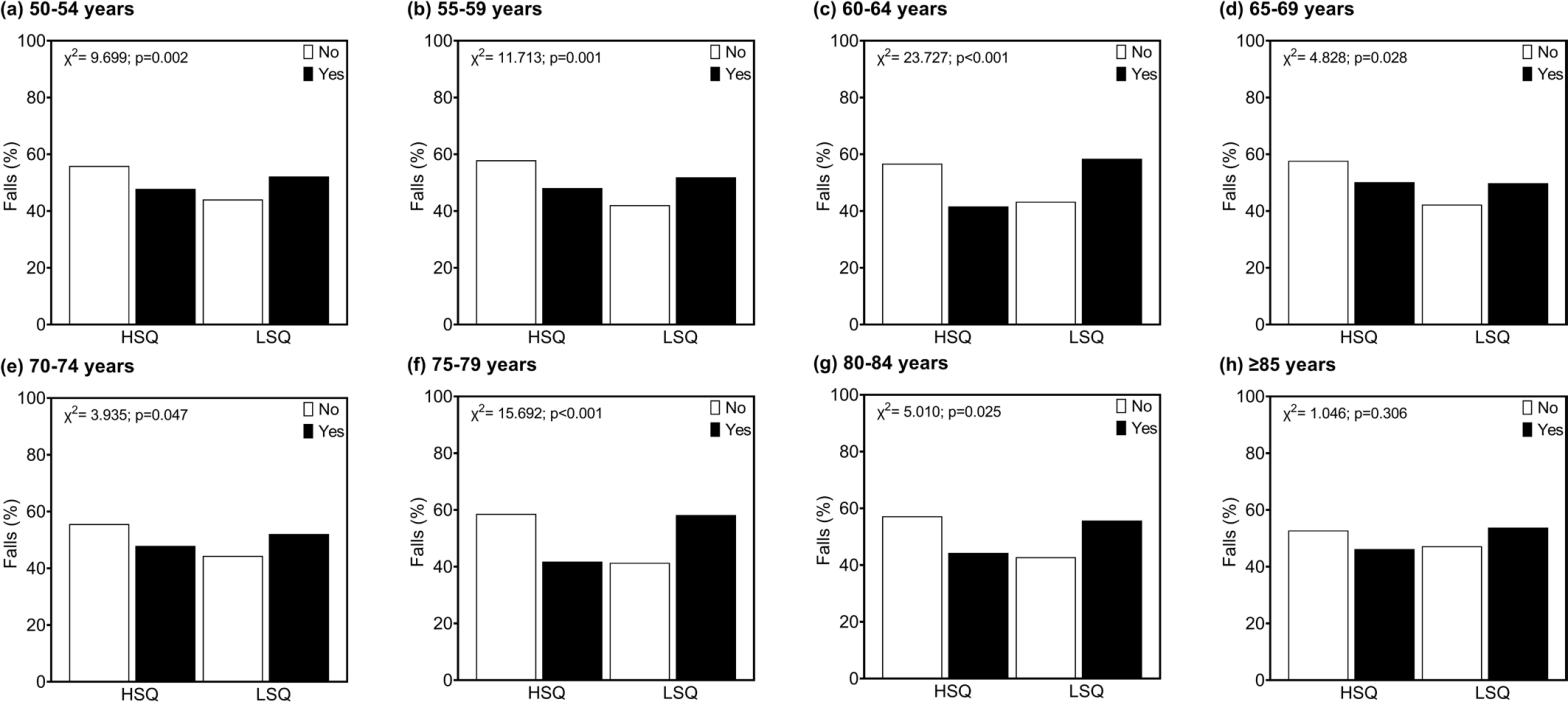
Association between participants with high self-reported sleep quality (HSQ) and low self-reported sleep quality (LSQ) with falls grouped by half-decade of life.

Legend: χ^2^ = test statistic value; p = p-value; HSQ = High self-reported Sleep Quality; LSQ = Low self-reported Sleep Quality.

In Figure 2, it can be observed a significant association (p < 0.05) of participants classified as having high sleep quality and LSQ with the frequency of falls in all age groups, except for people ≥ 85 years old.

**Figure 3:**
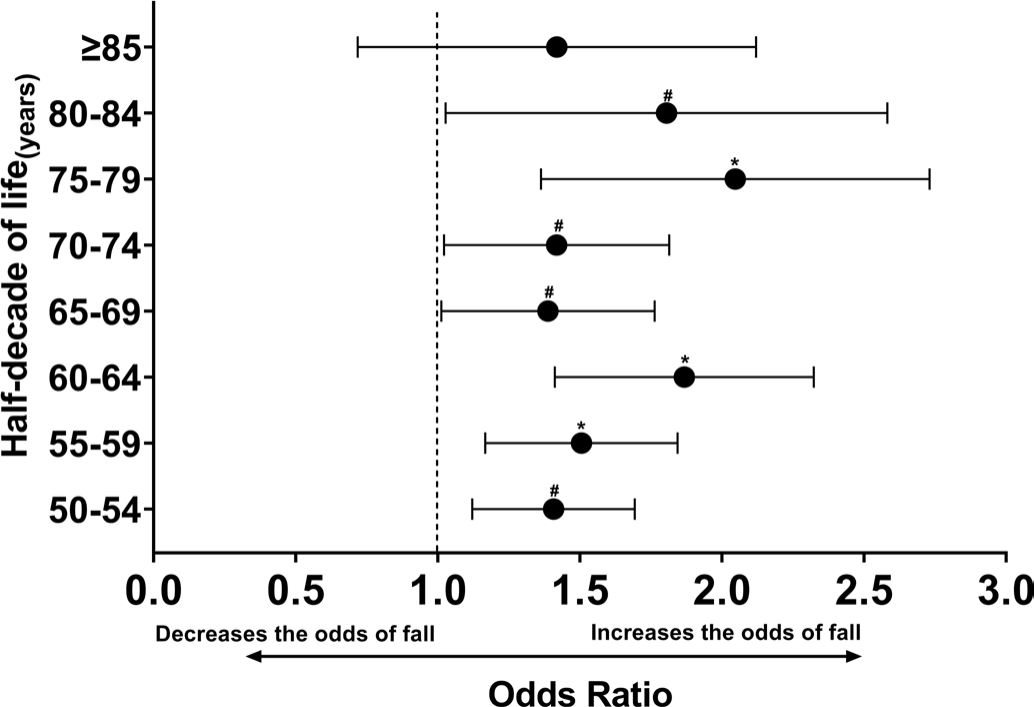
Odds ratio for low self-reported sleep quality to increase the chance of falls grouped by half-decade of life.

Legend: * = p < 0.001; ^#^ = p < 0.005.

In Figure 3, it can be observed that LSQ significantly elevates the odds of falls across age groups, of 50-54, 55-59, 60-64, 65-69, 70-74, 75-79, and 80-84 years. Additionally, there is an observed increasing trend in the OR for the occurrence of falls associated with LSQ every five years of life, up to the age of 84. These associations persist notwithstanding the consideration of sex and physical activity level as potential confounding variables.

## DISCUSSION

LSQ increased the falls in adults (from their 50s) and at every half-decade of life, until before the 80s. Even after controlling for confounding variables, including sex and physical activity level, LSQ remained associated with falls. To the best of our knowledge, this is the first study (nationally and internationally) to examine the OR of LSQ for fall occurrence every five years of life, highlighting LSQ as an important measure for fall monitoring in adults starting from their 50s. From our findings, encouraging and implementing strategies that foster good sleep hygiene can serve as an effective and accessible approach to enhancing the quality of life and safety of middle-aged and older individuals, contributing to an active and independent aging process. Furthermore, interventions should consider the overall quality of sleep and its potential influence on both upper and lower limb muscles.

Sleep disorders, such as the frequency of nocturnal awakenings, contribute to diminished sleep quality, consequently correlating with an increased susceptibility to falls^(20)^. This conclusion stems from an initial study published in 2007^(21)^, which established an association between sleep patterns and falls. The study encompassed 150 older hostel residents (aged 81 ± 8 years) and 150 internet survey participants (aged 70 ± 5 years). The study employed an extensively detailed sleep questionnaire, encompassing inquiries regarding the duration of sleep, the depth of sleep satisfaction, post-awakening alertness, diurnal napping frequency and duration, sleep quality assessment, as well as the quantification and characteristics of instances of nocturnal awakenings^(22)^. This facilitated a more intricate analysis of sleep aspects (nocturnal awakenings) that exert an influence on falls, a level of detail unattainable within our study due to its exclusive utilization of a Likert scale to assess overall sleep quality. Insomnia, another sleep disorder, exhibits a linkage with the falls among the older adults, as demonstrated by the data derived from the Study of Osteoporotic Fractures (SOF), conducted with a cohort exceeding 8,000 older females. This association held significance even after accounting for the confounding factor of insomnia medication usage (benzodiazepines)^(23)^. A comprehensive investigation involving a cohort exceeding 3,000 older American males concluded that, regardless of the influence of confounding variables, both subjectively and objectively assessed sleep disruptions exhibited an association with the susceptibility to falls among the older male demographic^(24)^. Daytime sleepiness (>10), sleeping 5 hours or less, nocturnal hypoxemia, and sleep efficiency were associated with a greater fall occurrence.

Even sleep aspects that do not inherently constitute sleep disorders, such as sleep duration, exhibit an affiliation with falls occurrences. This observation is underscored by findings from a dataset encompassing 1,542 community-dwelling individuals aged ≥ 68 years in Spain, collected through telephone interviews^(25)^. Even after adjustment for confounding variables such as lifestyle factors, health status, comorbidities, and both nocturnal and diurnal sleep-related complaints, individuals who slept for ≥ 11 hours, in comparison to those who slept between 7–8 hours, exhibited an elevated likelihood of experiencing recurrent falls. Furthermore, this relationship between sleep duration and fall incidents is particularly pronounced among females and the eldest subset of the population (>75 years). A random-digit dial telephone survey conducted with 971 females and 555 males living in northern California aged 64 to 99 years^(26)^, revealed that females being unmarried, living alone, having income less than $15,000 per year, difficulty walking, having more than one chronic medical condition, history of cardiovascular disease, hypertension, arthritis, sensory impairment, psychological difficulties, and nighttime sleep problems are all associated with falls. Nighttime sleep problems remain associated with falls even when controlling for all other risk factors for falling. A wide age range (18–89 years) data (1,334 subjects) from a rural area^(27)^ revealed that aging was associated with earlier sleep time and shorter sleep duration, and females reported longer and later sleep, but a poorer sleep quality than males.

A population-based data from the Korean Community Health Survey involving 201,700 participants ≥ 19 years old revealed that fallers and poor sleepers were more frequently observed in older adults (≥ 60 years) than in young (19-39 years) and middle-aged adults (40-59 years); poor sleeper was more prevalent in fallers than in non-fallers (44.0% vs 29.9%, p < 0.001). Compared to good sleep quality, poor sleep quality was significantly associated with an increased fall occurrence^(28)^.

Previous studies linked sleep duration with falls. However, this association was confirmed when 212,829 participants were analyzed in a meta-analysis^(27)^: short and long sleep duration were significantly associated with falls, being characterized by an ‘U-shaped’ curve (sleeping 7–8 h per day presented the lowest falls occurrence). The association between self-reported sleep and falls was studied prospectively (7.6 years) with 157,306 females from the Women’s Health Initiative^(27)^. Sleep ≤ 5 hours or ≥ 10 hours increased the odds of recurrent falls, together with LSQ, insomnia, and more sleep disturbances (even when data were adjusted to comorbidity, medications, and physical function)^(27)^.

When we check the trend between the different groups over half a decade, we can identify different behaviors in the percentage of falls, sleep, and physical activity. The percentage of individuals who experienced falls (21% to 24%) and those who did not (76% to 78%) was similar across the age groups grouped by half-decade of life. We found a study with a similar objective and measures to ours, but carried out with a sample of 150 hostel participants and 150 internet users, with an average age of 70 ± 5 years in the city of Sydney^(29)^. This study had a higher percentage of falls in the previous year than our study (44% for hostel participants and 41% for internet respondents). Another study with a similar objective and measures to ours was carried out in the city of Sunnyvale, California, with 1,526 individuals aged 64 to 99 years^(26)^. However, the prevalence of falls of 19% was close to that found by our study. Regarding self-reported sleep quality, there was an apparent tendency to stabilize across age groups grouped by half-decade of life. Approximately half of the studied sample had LSQ (44% to 49%), while the other half had HSQ (51% to 56%) for all the age groups grouped by half-decade of life. Interestingly, study carried out with 259 people living in the city of São Paulo-Brazil, showed trends in the “bad quality of sleep” different from our study. In this study, an apparent trend towards an increase in the percentage of the cross-age groups grouped by decade of life was found in older adults who had chronic pain (60–69 = 48%, 70–79 = 62%, and +80 = 73%)^(30)^. However, the same study showed a tendency for a decrease in the percentage of “bad quality of sleep” across the age groups grouped by decade of life in older adults who had no chronic pain (60–69 = 44%, 70–79 = 37%, and +80 = 35%)^(30)^. It is worth mentioning that this study is less representative of the Brazilian population. However, these results indicate that checking the presence of chronic diseases in these studies may be important. Returning to the previously mentioned study, they found a lower percentage of “poor or very poor sleep quality” than our study (15% for hostel participants and 26% for internet respondents). Regarding physical activity, an apparent trend of increasing sedentary classification in aging older adults across half-decade age groups emerged^(21)^. We did not find studies that reported sleep quality, fall occurrences, and physical activity across each half-decade of life within age groups with an identical measure, and as representative of a population as our study, enabling a more direct comparison. Arising from these discrepancies, we can hypothesize that the factors influencing falls may have distinct effects across various populations. Thus, it becomes important to evaluate the factors linked to falls and sleep in each population.

The current investigation has several strengths. As far as we know, this is the first study to examine the OR of LSQ for fall occurrence every five years of life. Another strength is the sample size, which covered a large range of ages and characteristics seldom covered in studies with older adults, amplifying the generalizability of our findings. Notwithstanding the promising outcomes garnered in this investigation, some limitations warrant consideration. The cross-sectional design employed herein precludes the inference of causation. Another limitation pertains to the sex imbalance, with a higher representation of females than males, which may influence the findings. Additionally, we highlight the potential for respondent bias.

Based on our findings, the promotion and implementation of strategies aimed at fostering proper sleep hygiene emerge as effective and accessible approaches to enhance the quality of life and safety of middle-aged and older adults, contributing to an active and independent aging process. Furthermore, interventions should consider the overall quality of sleep and its potential influence on both upper and lower limb muscles, paving the way for a comprehensive approach to improving the physical and functional well-being of this population. These results not only carry significant practical implications but also point toward future research areas that may provide additional insights into underlying mechanisms and more effective intervention strategies to reduce falls in this specific demographic group.

## CONCLUSION

Our study supports the hypothesis that LSQ is significantly associated with an increased occurrence of falls in adults aged >50 years, regardless of sex and physical activity level as confounding variables. LSQ among middle-aged adults, starting from their 50s, holds promise as a potential predictor for falls throughout each successive half-decade of life, up until just before reaching the 80s. It is important to note that for participants over 85 years old, there was no significant association. These results highlight the need to educate individuals about the benefits of sleep care and enable them to enjoy healthy aging.
